# Correction: G3BP–Caprin1–USP10 complexes mediate stress granule condensation and associate with 40S subunits

**DOI:** 10.1083/jcb.20150802809202019c

**Published:** 2019-12-18

**Authors:** Nancy Kedersha, Marc D. Panas, Christopher A. Achorn, Shawn Lyons, Sarah Tisdale, Tyler Hickman, Marshall Thomas, Judy Lieberman, Gerald M. McInerney, Pavel Ivanov, Paul Anderson

Vol. 212, No. 7, March 28, 2016. 10.1083/jcb.201508028.

The authors identified that some results in their article were based on the use of flawed constructs, as described in their recent Letter “Phosphorylation of G3BP1-S149 does not influence stress granule assembly” (Panas et al., 2019. *J. Cell Biol.* 10.1083/jcb.201801214). Specifically, the authors recently identified unintended mutations in the full-length pEGFP-C1-G3BP1-S149A and S149E constructs and the 1–168 truncations of GFP-G3BP1-S149A and S149E that confound the interpretation of experiments probing the effects of the G3BP-S149 phosphorylation event. The full-length versions of these constructs were generously provided by Dr. Jamal Tazi and previously used in other work (Tourriere et al., 2003. *J. Cell Biol.* 10.1083/jcb.200212128).

The figures representing data acquired using the flawed constructs are:

Fig. S1: Diagram depicting the S149A/E constructs (other constructs are unaffected)

Fig. 2:B, lanes 4 and 5; and C

Fig. 7:A, lanes 4, 5, 10, and 11

Fig. 8:B, lanes 6, 7, 12, and 13; and C

The results presented in these figures are unreliable and the authors apologize to the community for sharing these flawed data. The conclusions that the phosphomimetic G3BP-S149E mutant fails to rescue stress granule formation (Fig. 2, B and C), that coimmunoprecipitation of 40S proteins is not inhibited by G3BP mutation of S149 (Fig. 7 A), and that G3BP-S149E preferentially binds USP10 relative to Caprin1 (Fig. 8, B and C) are not reliable. The finding that the phosphomimetic G3BP-S149E mutant fails to rescue stress granule formation was confirmatory of prior work (Tourriere et al., 2003. *J. Cell Biol.* 10.1083/jcb.200212128).

However, other major conclusions in the study were reached independently of the flawed constructs. The conclusions that G3BP binds 40S ribosomes through its RGG region and is essential for stress granule condensation, stress granule condensation is distinct from polysome disassembly, and the condensation process is regulated by competitive Caprin1/USP10 binding to G3BP still stand firmly. In the article, the authors suggested that G3BP-S149 phosphorylation alters the outcome of stress granule formation by preferentially promoting the binding of G3BP to USP10 relative to Caprin1, but the authors now know that the S149E construct instead failed to rescue stress granule formation because of the unexpected S99P mutation (as shown in the Letter [Panas et al., 2019. *J. Cell Biol.* 10.1083/jcb.201801214]).

*JCB* editors appreciated the authors’ willingness and initiative to correct the published record. Because only a small part of the article reports flawed data as a result of a genuine error, the editors decided that correction of *JCB*’s published record was best served through a detailed correction notice. The editors’ assessment is that the paper’s major findings and contribution to the field remain reliable and that, upon removal of the flawed data, the experiments shown remain sound and well controlled.

The article text has been revised to omit the flawed data, its interpretation, and its discussion. The abstract has been edited. The title has not been changed. Figs. 2, B and C; 7 A; 8 B; and S1 and associated legends were edited to remove the parts of the panels pertaining to the flawed constructs. Fig. 8 C and its legend were deleted as (1) in the Results section, the only text that referenced panel C data pertained to flawed data and was deleted; (2) after removal of the flawed data from panel C, the remaining data were controls that are also present in the immunoblot shown in panel B; and (3) removal of panel C does not affect the major conclusions drawn from Fig. 8 about the selective association of G3BP with dissociated 40S ribosomal subunits based on the non-flawed data.

The previous, uncorrected version of the article and a marked-up PDF detailing the changes are available as supplemental files to the article. The HTML and PDF versions of this article have been corrected.

## Rockefeller University Press

